# Vaginal Microbiota Changes During Estrous Cycle in Dairy Heifers

**DOI:** 10.3389/fvets.2020.00371

**Published:** 2020-07-03

**Authors:** Juan J. Quereda, Marta Barba, María Lorena Mocé, Jesús Gomis, Estrella Jiménez-Trigos, Ángel García-Muñoz, Ángel Gómez-Martín, Pedro González-Torres, Belén Carbonetto, Empar García-Roselló

**Affiliations:** ^1^Microbiological Agents Associated With Animal Reproduction (ProVaginBio), Faculty of Veterinary Sciences, University Cardenal Herrera CEU, CEU Universities, Valencia, Spain; ^2^Microomics Systems S.L., Barcelona, Spain

**Keywords:** 16S metagenomics, vaginal microbiota, bovine, *Lactobacillus*, estrus, diestrus

## Abstract

The vaginal microbiota plays an important role in the health of dairy cattle, and it could be manipulated for the prevention and treatment of reproduction-related infections. The present study profiles and compares the vaginal microbiota of healthy dairy heifers during the estrous cycle focusing the results in follicular (estrus) and luteal (diestrus) phases using 16S rRNA sequencing of the V3–V4 hypervariable region. Twenty 13–16-months-old virgin dairy heifers from a single farm were included in this study. Vaginal swabs and blood samples were obtained during estrus (6–8 h before artificial insemination) and diestrus (14 days after insemination). Estrus was evaluated by an activity monitoring system and confirmed with plasma progesterone immunoassay. Results showed that the taxonomic composition of the vaginal microbiota was different during the follicular and luteal phases. At the phylum level, the most abundant bacterial phyla were Tenericutes, Firmicutes, and Bacteroidetes which comprised more than 75% of the vaginal microbiota composition. The next more abundant phyla, in order of decreasing abundance, were Proteobacteria, Actinobacteria, Fusobacteria, Epsilonbacteraeota, and Patescibacteria. Together with Tenericutes, Firmicutes, and Bacteroidetes represented more than 96% of the bacterial composition. *Ureaplasma, Histophilus, f_Corynebacteriaceae, Porphyromonas, Mycoplasma, Ruminococcaceae UCG-005*, were the most abundant genera or families. The results also showed that the vaginal microbiota of dairy heifers was non-lactobacillus dominant. The genus *Lactobacillus* was always found at a low relative abundance during the estrous cycle being more abundant in the follicular than in the luteal phase. Despite more research is needed to explore the potential use of native vaginal microbiota members as probiotics in dairy heifers, this study represents an important step forward. Understanding how the microbiota behaves in healthy heifers will help to identify vaginal dysbiosis related to disease.

## Introduction

Vaginal microbiota plays a key role in reproductive health and disease. Healthy human vaginal microbiota has been well-characterized. It is characterized by a low bacterial diversity with *Lactobacillus* spp. being the most abundant species with over 90% relative abundance on total bacteria (1). *Lactobacillus* spp. have been shown to play a key protective role by lowering the environmental pH through lactic acid production, by producing bacteriostatic and bactericidal compounds and through competitive exclusion (2, 3). Re-establishing a healthy *Lactobacillus*-dominant state through probiotic inoculation is an antibiotic-free strategy that has been well-described in human medicine (1). The abundance of *Lactobacillus* spp. in the bovine reproductive tract has been shown to be low (4, 5). However, the use of lactic acid bacteria as a probiotic treatment in the reproductive tract has been proved to confer protection against bacterial infections in dairy cows (6–9).

Uterine and vaginal cattle microbiota have been determined to contain higher diversity compared to human vaginal microbiota (4, 10). Nevertheless, Firmicutes were observed to be frequent and abundant in both (5, 11, 12). Despite the efforts that have been done to study the vaginal microbiota of dairy and beef cows (5, 10, 13, 14), there is scarce knowledge on the vaginal microbiota of healthy heifers (12). Understanding the influence of the estrous cycle on the reproductive microbiota is of the most importance since it will allow the identification of new therapeutic antibiotic-free strategies to treat infections during reproduction protocols. Currently, there is a knowledge gap describing the effect of the estrous cycle on cattle vaginal microbiota. Ault and colleagues have described that the diversity of the microbiota during luteal phase significantly decreases as the reproductive tract prepares for pregnancy (10). The same authors described differences in Verrucomicrobia, Fusobacteria, and Tenericutes abundances between pregnant and non-pregnant cows (14). However, the diversity and composition of the microbiota during estrus (high concentration of estrogen) was not evaluated in these studies. To the best of our knowledge, the variation in the vaginal microbiota during the estrous cycle in Holstein-Friesian dairy heifers in Europe using new generation sequencing approaches has not been studied yet. Therefore, this study aimed to describe the diversity and composition of the microbiota at the caudal vagina in healthy dairy heifers using a 16S rRNA based sequencing approach. Moreover, the vaginal microbiota in follicular (estrus) and luteal (diestrus) phases were compared. Our hypothesis was that vaginal microbiota was different depending on the ovarian cycle phase. In addition, we hypothesized that bovine vaginal microbiota was non-lactobacillus dominant.

## Materials and Methods

### Ethics Statement

Animal care and use protocols were approved by the Dirección General de Agricultura, Ganadería y Pesca-Generalitat Valenciana committee under protocol number 2017/VSC/PEA/00245 dated 22/12/2017.

### Farm Management and Animals

Twenty 13–16-months-old healthy virgin dairy heifers from a single farm located at Bétera (Valencia, Spain) were sampled between September and December 2018 months. Heifers were fed once daily a total mixed ration (TMR) consisting of barley straw, rapeseed meal, corn meal, orange pulp, salt, calcium carbonate, and mineral and vitamin corrector. Diets were formulated for meet nutrient requirements according to the NRC (15). Heifers were administered cloprostenol (Cyclix Bovino®, Virbac, España) injection to promote lysis of corpus luteum and estrus synchronization Heat was evaluated using an activity monitoring system (Nedap Smarttag Neck®) and confirmed by vaginal mucus discharge and standing heat. Physiological heat was considered when these three criteria were met.

### Vaginal Sample Collection

One vaginal swab sample was obtained during estrus (10–12 h after heat confirmation) and one during diestrus (14 days after insemination) in each heifer. Contamination was avoided by thoroughly cleaning with water and neutral soap the vulvar area and attaching the tail. Care was taken to avoid the moment of defecation while sampling. Then the vulva was opened by one operator and the second operator opened the sterile DNAse free cotton swab (Deltalab® ref 300263) sterilely and introduced it in the vaginal tract via the opened vulva, without touching the vaginal wall until the point of sampling at the caudal vagina. Samples were obtained by gently swabbing of the vaginal wall for 30 s. Then it was extracted carefully via the same methodology, avoiding contact with the vulva, and it was introduced in the transport tubes. The methodology was followed as previously described by Nugyere et al. (16). Swabs were stored at −80°C.

### Blood Collection and Progesterone Determination

Blood samples were collected the same day of vaginal swab collection from the middle caudal vein into heparinized vacutainer 5-mL tubes. The tubes were immediately centrifuged for 15 min at 3,000 × g. Plasma samples were frozen at −80°C for progesterone testing by solid-phase, competitive chemiluminescent enzyme immunoassay (Immunlite® 1000 Immunoassay System, Siemens Healthineers, Madrid, Spain). Progesterone concentration <1 ng/mL defined estrus and progesterone concentration >1 ng/mL defined diestrus. Mean and SD progesterone concentration in estrus was 0.23 ± 0.06 ng/ml and in diestrus, 6.45 ± 1.61 ng/ml. Estrus was determined clinically using an activity monitoring system (Nedap Smarttag Neck®), and confirmed by observing vaginal mucus discharge and standing heat.

### Library Preparation and Sequencing

DNA was extracted from swab samples using the DNeasy PowerLyzer PowerSoil Kit (Qiagen, Hilden, Germany) following the manufacturer's instructions. The extraction tubes were agitated twice using Tissue lyser II (Qiagen, Hilden, Germany) at 30 Hz/s for 10 min. Mock community DNA was included as positive control for library preparation (Zymobiomics Microbial Community DNA, ZymoResearch, Irvine, CA, USA). Samples were amplified using 16S rRNA V3-V4 regions specific primers (V3-V4-Forward 5′-TCGTCGGCAGCGTCAGATGTGTATAAGAGACAGCCTACGGGNGGCWGCAG-3′, V3-V4-Reverse 5′-GTCTCGTGGGCTCGGAGATGTGTATAAGAGACAGGACTACHVGGGTATCTAATCC-3′).

The PCR was performed in 10-μL final volume with 0.2-μM primer concentration. The PCR included: 3 min at 95°C (initial denaturation) followed by 25 cycles: 30 s at 95°C, 30 s at 55°C, and 30 s at 72°C, and a final elongation step of 5 min at 72°C. PCR products were purified using AMPure XP beads (Beckman Coulter, Nyon, Switzerland) with a 0.9 × ratio according to the manufacturer's instructions. PCR products were eluted from the magnetic beads with 32 μL of Milli-Q water and 30 μl of the eluate were transferred to a fresh 96-well plate. The above-described primers contain overhangs allowing the addition of full-length Nextera barcoded adapters for multiplex sequencing in a second PCR step, resulting in sequencing ready libraries with ~450 bp insert sizes. In brief, 5 μL of the first PCR purified product were used as the template for a second PCR with Nextera XT v2 adaptor primers in a final volume of 30 μL using the same PCR mix and thermal profile as for the first PCR but with only eight cycles. Twenty-five microliters of the second PCR product were purified with SequalPrep normalization kit (Invitrogen, ThermoFisher Scientific, Waltham, MA, USA), according to the manufacturer's protocol. Libraries were eluted in 20 μL final volume and pooled for sequencing. The final pool was quantified by qPCR using Kapa library quantification kit for Illumina Platforms (Kapa Biosystems, SigmaAldrich, Saint Louis, MO, USA) on an ABI 7900HT real-time cycler (Applied Biosystems, ThermoFisher Scientific, Waltham, MA, USA). Sequencing was performed using Illumina MiSeq with 2 × 300 bp reads using and v3 chemistry with a loading concentration of 10 pM. In all cases, 15% of PhIX control libraries were used to increase the diversity of the sequenced sample. Negative controls included sample collection buffer, DNA extraction, and PCR amplification steps, PRC products after both PCR steps were visualized using an electrophoresis gel (1.5% agarose) with SYBR Safe (Applied Biosystems, ThermoFisher Scientific, Waltham, MA, USA). No visible bands were observed.

### Amplicon Sequences Analysis

Raw demultiplexed forward and reverse reads were processed using the following methods and pipelines as implemented in QIIME2 version 2019.4 with default parameters unless stated (17). DADA2 was used for quality filtering, denoising, pair-end merging and amplicon sequence variant calling (ASV, i.e., phylotypes) using qiime dada2 denoise-paired method (18). Q20 was used as quality threshold to define read sizes for trimming before merging (parameters: –p-trunc-len-f and –p-trunc-len-r). Reads were truncated at the position when the 75th percentile Phred score felt below Q20: 288 bp for forward reads and 226 bp for reverse reads. After quality filtering steps, average sample size was 18,778 reads (min: 3,547 reads, max: 46,269 reads) and 4,399 phylotypes were detected. ASVs were aligned using the qiime alignment mafft method (19). The alignment was used to create a tree and to calculate phylogenetic relations between ASVs using *qiime phylogeny fasttree method* (20). ASV tables were subsampled without replacement in order to even sample sizes for diversity analysis using *qiime diversity core-metrics-phylogenetic pipeline*. The smallest sample size was chosen for subsampling (21). Jaccrad, Bary Curtis and unweighted and weighted Unifrac distances were calculated to compare community structure. The following alpha diversity metrics were calculated: observed OTU number (i.e., richness) and Pielou's evenness index. Taxonomic assignment of ASVs was performed using a Bayesian Classifier trained with Silva database (i.e., 99% OTUs database) using the *qiime* feature-classifier classify-sklearn method (22). Phylotypes were filtered to discard contaminant Eukariota DNA-derived amplicons using Blast against the mentioned database with a 90% identity cutoff.

A total of 4,926,964 pair-end reads were obtained before quality filtering. After quality filtering and trimming the reads, 3,502,033 reads remained. After denoising steps, 2,018,591 reads retained. The paired-end reads were merged, leaving 1,259,313 reads. After the chimera filter, 1,189,122 reads over 40 samples were used for phylotype calling with DADA2 and 6,251 phylotypes were detected. Singletones and doubletones were also removed. Samples were subsampled up to 3,500 reads to even sample size and make quantitative comparisons. Sample 18F (heifer 18 at follicular phase) was discarded since not enough reads were obtained after filtering (i.e., <1,000 reads).

Mock community and negative controls were processed the same way as samples.

### Statistical Analysis

Differential abundance of taxa was tested using Mann-Whitney (or Kruskal Wallis) non-parametric test (23). After Kruskal Wallis, Conover's test with FDR Benjamini-Hochberg correction was added for pairwise comparison. Alpha diversity comparisons were performed using Kruskal-Wallis non-parametric test. Fligner-Killeen test was used to test homogeneity of variances. Unifrac distance matrices and ASV tables were used to calculate principal coordinates and construct ordination plots. The significance of groups in community structure was tested using Permanova. Distance-based Redundancy Analysis (dbRDA) was used to explore which variables constrained PCoA ordinations, including progesterone concentration. Model selection was done using stepwise forward direction and a permutation test. Permdisp test was used to identify location vs. dispersion effects. Significant threshold was set at *p* ≤ 0.05. BiodiversityR version 2.11-1, PMCMR version 4.3, RVAideMemoire version 0.9-7 and vegan version 2.5-5 packages of the R software package version 36.6.0 (www.R-project.org) were used.

## Results

### Diversity Analysis

Rarefication curves showed that the achieved sequencing depth and subsampling size were enough to observe the complete diversity present in the sampled microbial communities. A plateau was reached for richness and evenness metrics (Supplementary Figure 1).

No significant differences were observed in richness and evenness between follicular and luteal samples. However, richness variance was significantly higher in microbial communities in follicular compared to luteal phase samples (*P* < 0.016) (Supplementary Figure 2).

**Figure 1:**
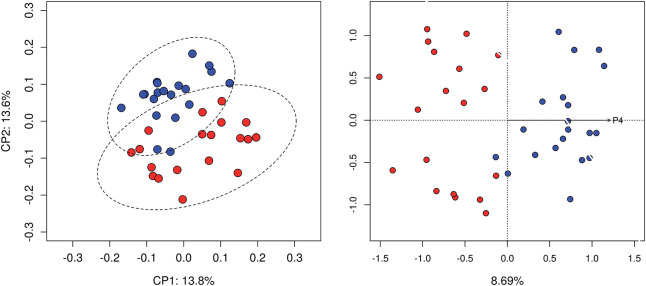
Beta diversity analysis. Principal coordinate analysis based on unweighted Unifrac distance matrix (left). dbRDA constrained ordination, 8.69% of the variance was explained by the concentration of progesterone (right). Samples at follicular (red) and luteal phases (blue) are compared.

Analysis of community structure using unweighted Unifrac distance showed differences between follicular and luteal samples (*P* < 0.05). No significant differences were detected when using weighted Unifrac distance. Moreover, dbRDA analysis showed that progesterone concentration constrained the ordination explaining part of the variance. 8.69% of the variance was explained by the concentration of progesterone (Figure 1). Analysis of community structure using weighted Unifrac did not show differences between follicular and luteal samples.

### Taxonomic Profiles

The variable region used in this study for amplicon sequencing allowed the detection of both Bacterial and Archaeal communities. As expected, Bacteria were more abundant than Archaea, accounting for 99.46% of reads. Bacteria and Archaea were detected in 100% (39/39) and 82% (32/39) of vaginal samples, respectively. It was decided to evaluate the taxonomic composition of samples by comparing the mean relative abundances of taxa in follicular and luteal phases but also in individual samples to explore inter-individual variability.

**Figure 2:**
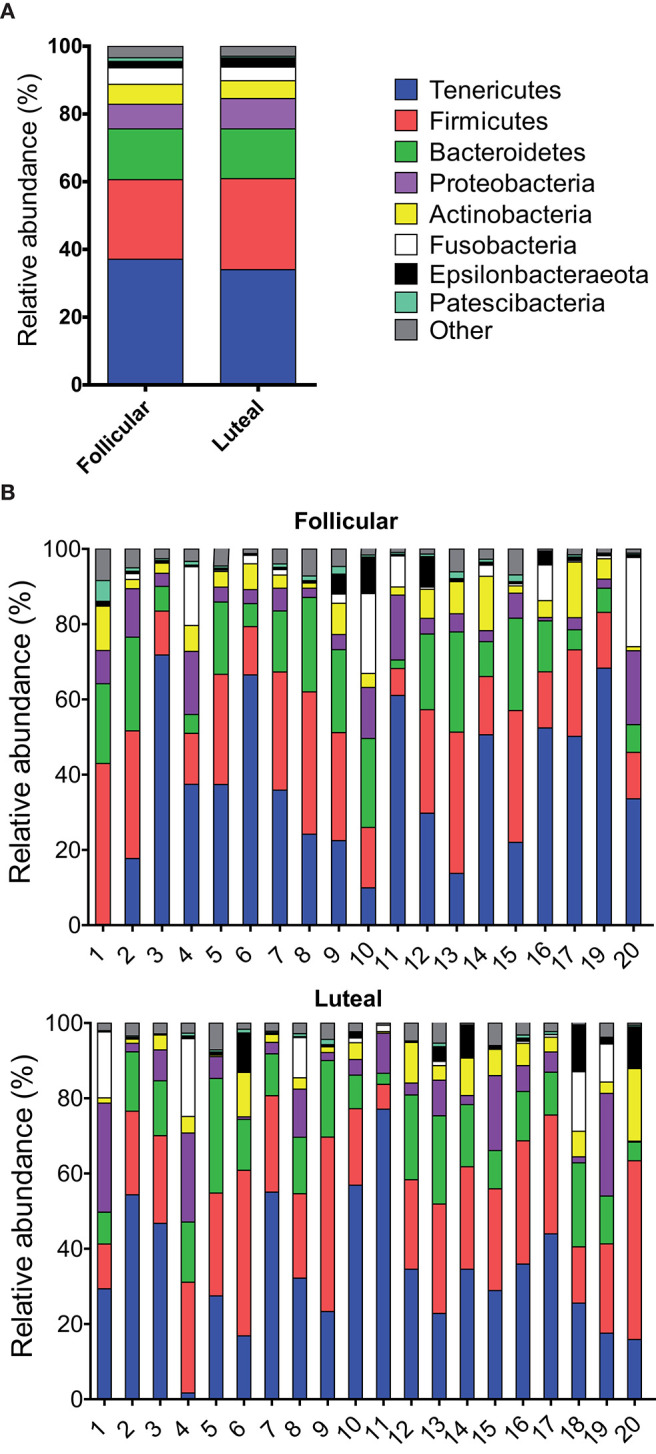
Relative abundance of phyla. Only taxa with a mean relative abundance >1.5% are shown. **(A)** Mean relative abundance in follicular and luteal phases. **(B)** Relative abundance in individual samples in follicular and luteal phases. Each column represents an individual sample. The numbers in the x axis correspond to each individual heifer.

Twenty-seven bacterial phyla were detected in vaginal samples during follicular and 23 bacterial phyla during the luteal phase. The most abundant bacterial phyla were Tenericutes, Firmicutes, and Bacteroidetes (35.6, 25.2, and 14.9%, respectively, calculated as the mean relative abundance, Figure 2), which represented more than 75%. The following abundant phyla, in order of decreasing abundance, were Proteobacteria, Actinobacteria, Fusobacteria, Epsilonbacteraeota and Patescibacteria, which together with Tenericutes, Firmicutes and Bacteroidetes spanned more than 96% of the bacteria taxa. Heifer 1 and heifer 4 presented negligible amounts of Tenericutes during the follicular and luteal phases, respectively (Figure 2).

Differences in taxonomic composition were observed between follicular and luteal phases. Regarding phyla, a decrease in the relative abundance of Chloroflexi, Deinococcus-Thermus, WPS-2 (*P* < 0.001), Cloacimonetes, Patescibacteria, and Planctomycetes (*P* < 0.05) were observed in luteal phase samples when compared to follicular samples (Supplementary Figure 3). In contrast, the relative abundance of the phylum Kiritimatiellaeota was higher in the luteal than in the follicular phase (*P* < 0.01) (Supplementary Figure 3).

**Figure 3:**
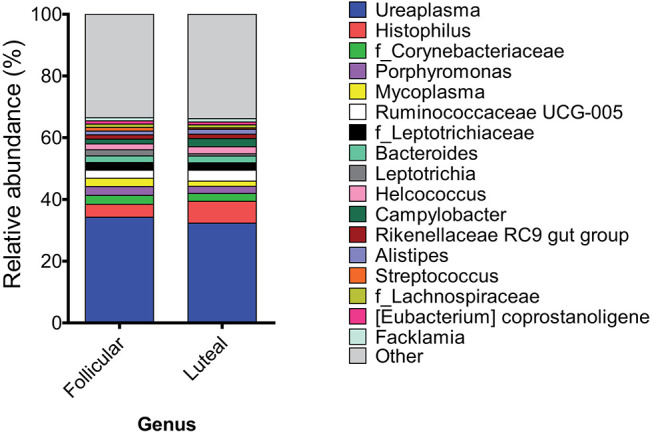
Mean relative abundance at the genus level in samples at follicular and luteal phases. Only taxa with a mean relative abundance >1.5% are shown.

At the genus or family levels (when the genus or subsequent taxa could not be assigned), 17 genera and 4 families showed relative abundance >1% (Figure 3, Table 1 and Supplementary Table 1). *Ureaplasma, Histophilus, f_Corynebacteriaceae, Porphyromonas, Mycoplasma, Ruminococcaceae UCG-005, f_Leptotrichiaceae, Bacteroides, Leptotrichia, Helcococcus, Campylobacter, Rikenellaceae RC9 gut group, Alistipes, Streptococcus, f_Lachnospiraceae, (Eubacterium) coprostanoligenes group*, and *Facklamia* were the most abundant genera or families.

**Table 1:** Genera or families (when the genus or subsequent taxa could not be assigned) in follicular and luteal phases with relative abundance >1%.

**Taxa**	**Follicular phase Mean relative abundance (%)**	**Luteal phase Mean relative abundance (%)**
*Ureaplasma*	34.2	32.3
*Histophilus*	4.2	7.1
*f_Corynebacteriaceae*	2.9	2.5
*Porphyromonas*	2.9	2.4
*Mycoplasma*	2.7	1.6
*Ruminococcaceae UCG-005*	2.6	3.5
*f_Leptotrichiaceae*	2.5	2.4
*Bacteroides*	2.1	2.2
*Leptotrichia*	2.0	0.7
*Helcococcus*	1.9	2.3
*Campylobacter*	1.6	2.7
*Rikenellaceae RC9 gut group*	1.4	1.4
*Alistipes*	1.2	1.6
*Streptococcus*	1.2	0.4
*f_Lachnospiraceae*	1.2	1.0
*[Eubacterium] coprostanoligene*	1.0	1.0
*Facklamia*	1.0	1.1
*Ruminococcaceae UCG-010*	1.0	1.7
*Peptoniphilus*	0.9	1.6
*Arcanobacterium*	0.4	1.2
*f_Ruminococcaceae*	0.9	1.2
*Christensenellaceae R-7 group*	0.9	1.2
*Prevotellaceae UCG-003*	0.6	1.1

Regarding genera or families, a significant decrease in the relative abundance of *Nocardioides, uncultured Porphyromonadaceae bacterium, Aerococcus, Lactobacillus, Coprococcus, Leptotrichia, Paracoccus, W5053, Puniceicoccus* (*P* < 0.05), *f_ Intrasporangiaceae, Proteiniphilum, Paraburkholderia tropica, Streptococcus, Acinetobacter, Pseudomonas* (*P* < 0.01), *Membranicola, Planktosalinus, Arcobacter, Guggenheimella, Fastidiosipila, Pelagibacterium, Marinobacterium, Arenimonas*, and *Thauera* (*P* < 0.001) was observed in luteal phase samples compared to the follicular phase samples (Supplementary Figure 4).

In contrast, the relative abundance of families *f_ Paludibacteraceae, f_Bacteroidales RF16 group (g_uncultured Paludibacter sp*), and Family XIII AD3011 group (*P* < 0.05) and genera *Turicibacter, Prevotellaceae UCG-003, Ruminococcaceae UCG-010, Solibacillus, Ruminobacter* (*P* < 0.05), *Romboutsia* (*P* < 0.01), *Psychrobacter*, and *Paeniclostridium* (*P* < 0.001) was higher in luteal phase samples (Supplementary Figure 5).

The genus *Lactobacillus* was detected in 37% (*n* 7/19) and 10% (*n* 2/20) of follicular and luteal samples, respectively. The genus *Lactobacillus* was always found at a low relative abundance (0.19–0.04%). Only *Ureaplasma, Ruminococcaceae UCG-005, Bacteroides* and *f_ Ruminococcaceae* were identified in all vaginal samples (Table 1 and Supplementary Table 1). Although *Ureaplasma* was the most abundant genus in most of the animals, this genus was almost absent in the heifer 1 in the follicular phase (relative abundance of 0.015%). Similarly, although the genus *Histophilus* was the second in terms of relative abundance in the dataset, it was not detected in heifer 1 and heifer 9 follicular phase samples and heifers 6, 12, 18, and 20 in luteal phase samples.

Regarding Archaea, only the phylum Euryarchaeota was observed (0.55% mean relative abundance) in 37% (*n* 7/19) and 75% (*n* 15/20) of follicular and luteal samples, respectively. Among the genera found, a high prevalence of *Methanobrevibacter* was observed, representing almost 85% of all Archaea.

## Discussion

The present study characterized the vaginal microbiota of Holstein-Friesian dairy heifers during the estrous cycle. This is the first time heifer's vaginal microbiota was analyzed in the same animals through the same reproductive cycle. High diversity of Bacteria and low diversity of Archaea were detected. The observed differences in the community structure of the vaginal microbiota associated with progesterone shifts during the estrous cycle were consistent with previous observations on human vaginal microbiota (Human Microbiome Project Consortium, 2012).

The most abundant bacterial phyla detected in dairy heifers vaginal microbiota were Tenericutes, Firmicutes, and Bacteroidetes, with over 75% relative abundance. Clemmons and colleagues have reported three dominant phyla in the vaginal microbiota of Angus breed cows in EEUU; Firmicutes (65.9%), Bacteroidetes (16.8%), and Proteobacteria (7.4%) with only 2.8% of Tenericutes (5). Laguardia-Nascimiento and colleagues have also observed that the three most abundant bacterial phyla in the vaginal microbial communities of brazillian Nellore cattle were Firmicutes (40–50%), Bacteroidetes (15–25%) and Proteobacteria (5–25%), but Tenericutes was not detected (12). These authors have suggested an intestinal origin for the vaginal microbes, explained by the anatomical proximity (5, 12). Ault and colleagues have previously described differences in the abundance of Tenericutes and Acidobacteria in pregnant and non-pregnant post-partum cows with a higher abundance of these phyla in non-pregnant cows (14).

The absence or lower abundance of the Tenericutes phylum in previous studies compared with the present study could be related to differences in breed, geographic location, diet, and age, among others (5, 12). The vaginal microbiota has already been shown to be highly dependent on the environmental conditions and host factors (11). Several studies have reported variations in the composition and structure of the human vaginal microbiota among women from diverse geographic locations, races, and ethnicities (24). Moreover, the impact of the diet (especially probiotics and prebiotics) on human vaginal microbial composition has been reported (25).

In this study, the most abundant genera were *Ureaplasma, Histophilus, f_Corynebacteriaceae, Porphyromonas, Mycoplasma, Ruminococcaceae UCG-005*, and *f_Leptotrichiaceae*. *Ureaplasma* presented a relative abundance higher than 30%. *Ureaplasma diversum* has been previously reported in bovine vaginal microbiota in several countries including Brazil (26, 27), France (28), Canada (29), Costa Rica (30), Argentina (31), Australia (32), and Spain (33). Particularly in Canada, *U. diversum* has been identified in the microbiota of the reproductive tract of cattle without clinical signs of granular vulvitis (29, 34). In Spain, the presence of *U. diversum* was related to granular vulvovaginitis and subclinical endometritis (33). Interestingly, the abundance of *Ureaplasma* has been reported to be high in the uterine microbiota of cows that failed to become pregnant after 200 days in milk (35). The dairy heifers included in the present study were healthy, without visible vulvar lesions and showed a mean fertility of 85% at first insemination in contrast with previous reports (36). More studies are needed to elucidate the effect of *Ureaplasma* groups on the reproductive performance of dairy cows. *Histophilus* was detected as the second most abundant genus in this study. The presence of *Histophilus* spp has already been reported to be part of the microbiota of male and female bovine genital tract and its presence does not imply any disease (37).

It is worth noticing that even if beta diversity analysis (unweighted unifrac) showed differences between microbial communities in luteal and follicular phases, the differences in taxonomic composition were only observed for low abundant taxa (even absent in most samples) and not for abundant groups as shown in Supplementary Figures 4, 5. Remarkably, the importance of low abundant microorganisms has been reported in other systems, such as the bacterial gut community (38). Low-abundant microbes may contain a pool of genes whose expression carries out metabolic processes important to the overall microbial community, for example enhancing or triggering the metabolic activity of more dominant members. Benjamino et al., reported that low-abundant bacteria that often do not belong to the core gut community are drivers of the hindgut bacterial community composition (38). Altogether, these results suggest that low abundant or rare taxa could play an important role in the vaginal microbiota of cows during the estrous cycle, however it remains unknown.

The genus *Lactobacillus* was found at a low relative abundance through the estrous cycle. These results were in agreement with previous studies that used culture-independent methods to characterize the vaginal microbiota of ruminants (4, 14). Here, differences in relative abundance throughout the estrous cycle were detected for 32 genera and 3 families. Importantly, the abundance of *Lactobacillus* was higher in the follicular than in the luteal phase. Otero and colleagues also described a decrease of *Lactobacillus* abundance related to the raise of progesterone concentration in cattle vaginal microbiota (39). It has also been shown that the concentration of sexual hormones (especially estrogen) is an important factor responsible for the observed temporal dynamics in the composition of the human vaginal microbiota (40). It is known that *Lactobacillus* spp. play a key role to keep the eubiosis of the human vaginal microbiota by inhibiting the proliferation of undesirable microorganisms (41). While *Lactobacillus* spp. are abundant in the human vaginal microbiota and scarce in cattle vaginal microbiota, other bacteria taxa may play this key role in cows. More research is needed to elucidate this possible role of other beneficial vaginal bacteria in ruminants.

Concerning Archaea, the only phylum observed was Euryarchaeota, and the major genus was *Methanobrevibacter*. These results are very similar to those found by Laguardia-Nascimento in brazillian Nellore cattle who suggested that the exposition of vaginal lumen to large numbers of *Methanobrevibacter* from the bovine gastrointestinal tract could explain the origin of this genus inside the vagina (12). Archaea is a highly diverse group of prokaryotes. However, the diversity of Archaea associated to mammals is low. For example, only representatives of the phylum Euryarchaeota are present in the human body. This phylum includes three species: *Methanobrevibacter oralis*, found mostly in the oral cavity, *Methanosphaera stadtmanae* found mostly in the gut and *Methanobrevibacter smithii*, found mostly in the gut and vagina. Despite the increasing information about archaeal genomes, structure, and function, much remains unknown and up to date there is no substantial evidence supporting pathogenic properties (42).

Even if in the present study, only heifers were studied to reduce possible variation due to stage of development and reproductive manipulation history, the composition of the bacterial communities studied here were highly heterogeneous between animals (Figure 2B) as previously described by other authors in the bovine uterus (43, 44). These results are in agreement with previous studies in cows and heifers where individual variation was more important than age or pregnancy status (12). Variation among individuals, including anatomical and physiological differences, may also influence the microbiota of the reproductive tract (12).

## Conclusion

The most abundant bacterial phyla in the vaginal microbiota of Holstein Friesian dairy heifers were shown to be Tenericutes, Firmicutes, and Bacteroidetes which comprised over 75% relative abundance of bacteria. The composition of microbial communities was observed to be highly dispersed between animals and over the estrous cycle. Differences in relative abundance throughout the estrous cycle were observed for 32 genera and three families, even for the genus *Lactobacillus* which was not among the most abundant genera.

Studying the differences in the vaginal microbiota composition and diversity in healthy animals during the estrous cycle is important to later compare these results with results on non-healthy animals to reveal microbial biomarkers of disease. The discrimination of beneficial bacterial groups can also lead to the utilization of probiotic-based treatments. Moreover, if a relationship is to be discovered between the vaginal microbial composition of healthy animals and fertility rates in cows, biomarkers for reproduction can also be revealed. This study represents a first important step forward in this direction by characterizing abundant and non-abundant but fluctuating taxa in dairy heifers vaginal microbiota. Moreover, the heterogeneity of community composition between individuals was confirmed enhancing the need of larger experimental sizes for future studies.

## Data Availability Statement

The data discussed in this publication have been deposited in the NCBI Sequence Read Archive (http://www.ncbi.nlm.nih.gov/Traces/sra/) and are accessible through accession number PRJNA629084.

## Ethics Statement

Animal care and use protocols were approved by the Dirección General de Agricultura, Ganadería y Pesca-Generalitat Valenciana committee under protocol number 2017/VSC/PEA/00245 dated 22/12/2017. Written informed consent was obtained from the owners for the participation of their animals in this study.

## Author Contributions

JQ and EG-R contributed to the conception and design of the study. JG and ÁG-Mu performed the sample collection. MB, ÁG-Ma, EJ-T, JQ, and EG-R performed the microbiological laboratory methodology. PG-T and BC performed the metagenomic methodology. MM, PG-T, and BC performed the statistical analysis. JQ, EG-R, and MB wrote the sections of the manuscript. All authors contributed to the article and approved the submitted version.

## Conflict of Interest

PG-T and BC were employed by the company Microomics Systems S.L. The remaining authors declare that the research was conducted in the absence of any commercial or financial relationships that could be construed as a potential conflict of interest.
