# Supplementary material for: Vaginal Microbiota Changes During Estrous Cycle in Dairy Heifers
Source: Front Vet Sci. 2020 Jul 3;7:371. doi: 10.3389/fvets.2020.00371 (PMC7350931; doi:10.3389/fvets.2020.00371)
Supplement: Supplementary file 1 [file Data_Sheet_1.docx]

Supplementary Material

## Supplementary Figures

**SUPPLEMENTARY FIGURE 1.** Rarefication curves showing richness (A) and evenness metrics (B). Follicular (red) and luteal phases (blue) are compared.

**
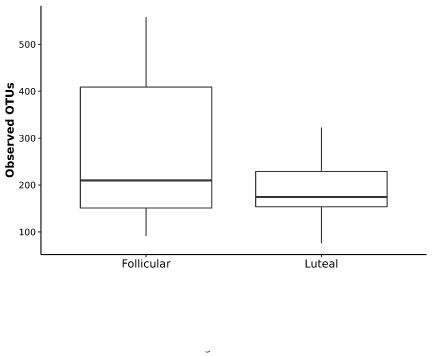
**

**SUPPLEMENTARY FIGURE 2**. Richness of vaginal microbial communities in follicular and luteal phases.


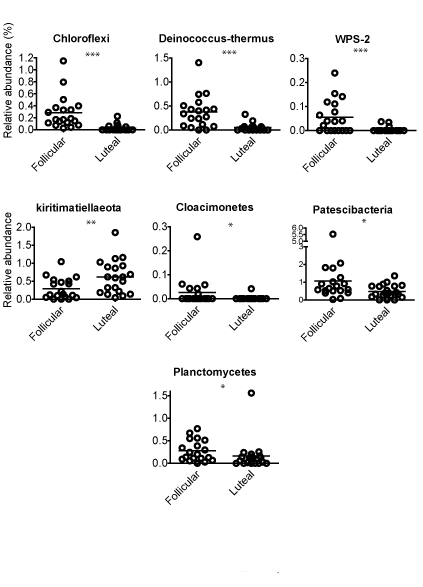


**SUPPLEMENTARY FIGURE 3**. Phyla with significant differences in relative abundances between follicular and luteal phases. Each dot represents an individual sample. Only taxa with a mean relative abundance > 0.05% are shown. *P < 0.05, **P < 0.01, ***P < 0.001.


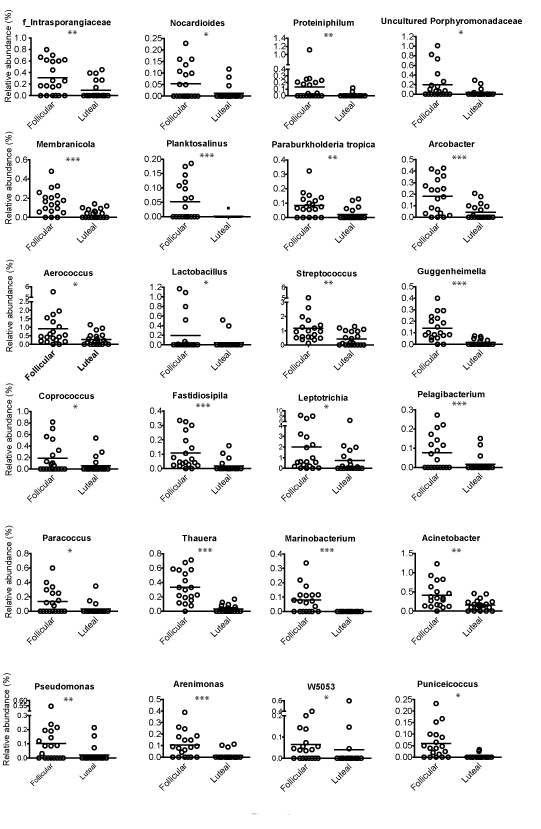


**SUPPLEMENTARY FIGURE 4**. Genera or families (when the genus or subsequent taxa could not be assigned) showing significant differences in relative abundances between follicular and luteal phases. Only taxa with a significant decrease in luteal when compared to follicular phase and only taxa with a mean relative abundance > 0.05 % are shown. Each dot represents the value for one heifer. *P < 0.05, **P < 0.01, ***P < 0.001.


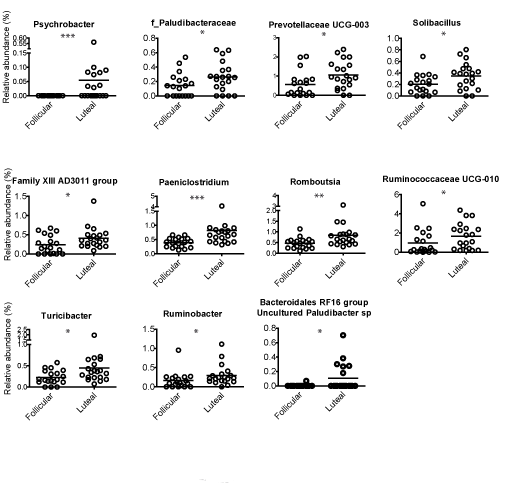
**SUPPLEMENTARY FIGURE 5**. Genera or families (when the genus or subsequent taxa could not be assigned) showing significant differences in relative abundances between follicular and luteal phases Only genera with a significant increase in luteal when compared to follicular phase and only taxa with a mean relative abundance > 0.05 % are shown. Each dot represents the value for one heifer.

## Supplementary Table

**Supplementary Table 1.** Genera or families (when the genus or subsequent taxa could not be assigned) in follicular and luteal phases with relative abundance > 0.2%

| Taxa | Follicular phase  Mean relative abundance (%) | Luteal phase  Mean relative abundance (%) |
| --- | --- | --- |
| *Ureaplasma* | *34.2* | *32.3* |
| *Histophilus* | *4.2* | *7.1* |
| *f_Corynebacteriaceae* | *2.9* | *2.5* |
| *Porphyromonas* | *2.9* | *2.4* |
| *Mycoplasma* | *2.7* | *1.6* |
| *Ruminococcaceae UCG-005* | *2.6* | *3.5* |
| *f_Leptotrichiaceae* | *2.5* | *2.4* |
| *Bacteroides* | *2.1* | *2.2* |
| *Leptotrichia* | *2.0* | *0.7* |
| *Helcococcus* | *1.9* | *2.3* |
| *Campylobacter* | *1.6* | *2.7* |
| *Rikenellaceae RC9 gut group* | *1.4* | *1.4* |
| *Alistipes* | *1.2* | *1.6* |
| *Streptococcus* | *1.2* | *0.4* |
| *f_Lachnospiraceae* | *1.2* | *1.0* |
| *Eubacterium] coprostanoligenes group* | *1.0* | *1.0* |
| *Facklamia* | *1.0* | *1.1* |
| *Ruminococcaceae UCG-010* | *1.0* | *1.7* |
| *Aerococcus* | *0.9* | *0.3* |
| *Christensenellaceae R-7 group* | *0.9* | *1.2* |
| *f_Ruminococcaceae* | *0.9* | *1.2* |
| *Peptoniphilus* | *0.9* | *1.6* |
| *Corynebacterium 1* | *0.8* | *0.2* |
| *Prevotellaceae UCG-004* | *0.6* | *0.7* |
| *Phascolarctobacterium* | *0.6* | *0.6* |
| *Prevotellaceae UCG-003* | *0.6* | *1.1* |
| *Methanobrevibacter (Archaea)* | *0.6* | *0.4* |
| *uncultured Porphyromonadaceae bacterium* | *0.5* | *0.7* |
| *Romboutsia* | *0.5* | *0.8* |
| *Anaerococcus* | *0.4* | *0.7* |
| *Moheibacter* | *0.4* | *0.0* |
| *Alloprevotella* | *0.4* | *0.6* |
| *Akkermansia* | *0.4* | *0.6* |
| *Candidatus Saccharimonas* | *0.4* | *0.4* |
| *f_Muribaculaceae* | *0.4* | *0.4* |
| *Acinetobacter* | *0.4* | *0.2* |
| *Arcanobacterium* | *0.4* | *1.2* |
| *Paeniclostridium* | *0.4* | *0.8* |
| *f_Saprospiraceae* | *0.4* | *0.1* |
| *Ruminococcaceae UCG-013* | *0.4* | *0.2* |
| *Trueperella* | *0.4* | *0.5* |
| *Truepera* | *0.4* | *0.1* |
| *Clostridium sensu stricto 1* | *0.4* | *0.5* |
| *Corynebacterium* | *0.4* | *0.3* |
| *Treponema 2* | *0.4* | *0.3* |
| *Fusobacterium* | *0.3* | *0.9* |
| *f_Prevotellaceae* | *0.3* | *0.4* |
| *Ruminococcaceae NK4A214 group* | *0.3* | *0.4* |
| *Ruminococcaceae UCG-014* | *0.3* | *0.1* |
| *Thauera* | *0.3* | *0.0* |
| *f_Intrasporangiaceae* | *0.3* | *0.1* |
| *uncultured Bacteroidales bacterium* | *0.3* | *0.6* |
| *f_Peptococcaceae* | *0.2* | *0.3* |
| *D_3__Bacteroidales* | *0.2* | *0.3* |
| *Family XIII AD3011 group* | *0.2* | *0.4* |
| *Atopostipes* | *0.2* | *0.6* |
| *Prevotella 9* | *0.2* | *0.0* |
| *Prevotellaceae UCG-001* | *0.2* | *0.3* |
| *Ruminococcaceae UCG-002* | *0.2* | *0.2* |
| *Turicibacter* | *0.2* | *0.4* |
| *Lachnospiraceae UCG-010* | *0.2* | *0.2* |
| *Solibacillus* | *0.2* | *0.3* |
| *uncultured Porphyromonadaceae bacterium* | *0.2* | *0.0* |
| *Lactobacillus* | *0.2* | *0.0* |
| *Lachnospiraceae NK4A136 group* | *0.2* | *0.3* |
| *Coprococcus 3* | *0.2* | *0.1* |
| *Ruminococcaceae UCG-009* | *0.2* | *0.2* |
| *D_3__Gastranaerophilales;D_4__uncultured bacterium;D_5__uncultured bacterium* | *0.2* | *0.2* |
| *Arcobacter* | *0.2* | *0.0* |
| *p-1088-a5 gut group* | *0.2* | *0.2* |
| *f_Prolixibacteraceae* | *0.2* | *0.0* |
| *Ruminobacter* | *0.2* | *0.3* |
| *[Eubacterium] nodatum group* | *0.2* | *0.2* |
| *Flavonifractor* | *0.2* | *0.2* |
| *Membranicola* | *0.2* | *0.0* |
